# Thermal radiation from subwavelength objects and the violation of Planck’s law

**DOI:** 10.1038/s41467-019-11287-6

**Published:** 2019-07-26

**Authors:** Juan Carlos Cuevas

**Affiliations:** Departamento de Física Teórica de la Materia Condensada and Condensed Matter Physics Center (IFIMAC), Universidad Autónoma de Madrid, Madrid, 28049 Spain

**Keywords:** Optics and photonics, Nanoscale materials, Nanoscale devices

## Abstract

Thermal radiation is a ubiquitous physical phenomenon that has been usually described with the help of Planck’s law, but recent developments have proven its limitations. Now, experimental advances have demonstrated that the far-field thermal radiation properties of subwavelength objects drastically violate Planck’s law.

Our understanding of the phenomenon thermal radiation has been traditionally based on Planck’s law^1^ and the concept of black body. This law sets upper limits for the amount of energy that an object can emit or exchange in the form of electromagnetic waves. However, due to the development of novel experimental techniques it has been possible to probe the limitations of this law in different situations^2^. Most of the recent work has been devoted to exploring the physics beyond Planck’s law in the near-field regime, i.e., when objects are separated by distances smaller than the thermal wavelength *λ*_Th_ set by Wien’s displacement law (~10 μm at 300 K). In particular, different experiments have demonstrated that due to the contribution of evanescent waves, the radiative heat transfer between two objects can largely exceed the black-body limit by bringing them sufficiently close^3–5^. In the far-field regime, when only propagating waves contribute, no big surprises are expected. However, very recently it has become clear that the thermal radiation properties of subwavelength objects can dramatically violate Planck’s law even in the far-field regime^6–8^, which opens new opportunities for the field of thermal radiation.

## Thermal emission of subwavelength objects

According to Planck’s law, the thermal emission of an object is incoherent, broad band, almost isotropic, and unpolarized. However, the application of concepts and techniques of nanophotonics has challenged this common wisdom^9^. In particular, nanophotonic structures have been designed to tune the thermal emission, including its spectral distribution^10^, polarization^11^, and angular dependence^11^. On the other hand, it has been predicted that the thermal emission of an object can drastically deviate from the expectations of Planck’s law when some of its dimensions are smaller than *λ*_Th_^12^. In this case, the total thermal emission could even overcome the black-body limit in some peculiar situations^12^. Deviations from Planck’s law were in fact reported a few years ago in an experiment on the thermalisation of an optical fibre thinner than *λ*_Th_, although the total thermal emission was found to be clearly below the black-body limit^13^. Apart from this notable exception, it has turned out to be very challenging to measure the thermal emission properties of subwavelength objects.

**Fig. 1:**
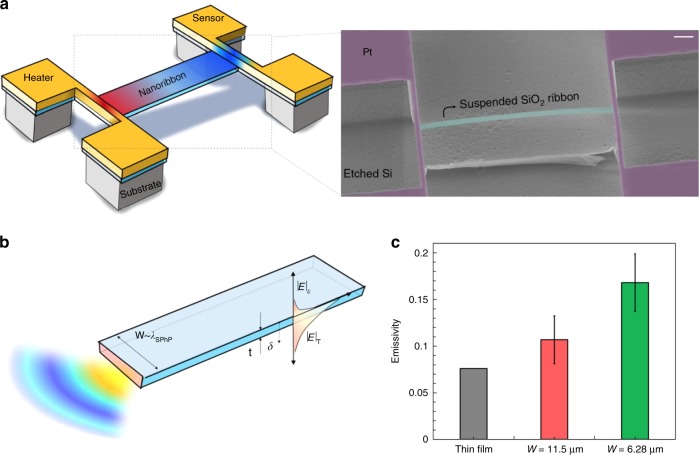
Measuring the thermal emissivity of SiO_2_ nanoribbons. **a** Schematic illustration and scanning electron microscope (SEM) image of the suspended thermal transport measurement micro-device with a SiO_2_ nanoribbon where one can see the suspended heater and sensor electrodes (Pt) and a nanoribbon across the electrodes^8^. The scale bar in the SEM image represents 30 μm. **b** Schematic illustration of a long SiO_2_ nanoribbon with rectangular cross-section with thickness *t* and width *W*. Here, *t* is smaller than the skin depth *δ* and the thermal wavelength *λ*_Th_, while *W* is comparable to the wavelength of the surface phonon polaritons *λ*_SPhP_. **c** Measured emissivity at room temperature for 100 nm-thick ribbons with thickness of 6.28 and 11.5 μm. This emissivity is compared to the computed result for an infinitely wide thin film of the same thickness. Reprinted from ref. ^8^ with permission from Springer Nature

In this context, Renkun Chen and colleagues have reported the development of a novel experimental platform to measure the thermal emissivity of an individual nanoscale object^8^. This platform makes use of a very sensitive thermometry to measure the thermal conductance of an object in combination with optical modelling to extract its thermal emissivity (Fig. 1a). This technique was used to investigate the thermal emission of nanoribbons made of a polar dielectric (SiO_2_) with a thickness of 100 nm, much smaller than both *λ*_Th_ and the skin depth of the material (Fig. 1b). The challenge in this case was to probe the thermal emission from an emitter, the nanoribbon, which has a very low emitting power (on the order of nW). In this case, the thermometry approach was used to measure the temperature across the suspended nanoribbons. This measurement, together with the knowledge of the thermal conductivity of the material, enabled the extraction of the radiative heat loss with the help of a thermal fin model. As a result, they could obtain the thermal emissivity of the object.

Bulk silica behaves like a good black body in the broad infrared range, except within the Reststrahlen band. By reducing the ribbon thickness below the skin depth, the authors managed to suppress the broadband emission in favour of the coherent, narrowband contribution coming from the surface phonon polaritons supported by this polar dielectric. The main finding of this work was the observation that the nanoribbons exhibit a higher emissivity (up to 8 times) than silica films of the same thickness (Fig. 1c). This enhanced emissivity was attributed to the anisotropic emission of these nanoribbons. In particular, as it has been recently predicted^6^, these nanoribbons emit thermal radiation very efficiently through the edges with a directional emissivity that overcomes the black-body limit. While in this case the total emissivity, integrated over the whole nanoribbon, is still clearly smaller than 1, the importance of these experiments lies in the fact that this is one of the few available techniques that is sensitive enough to characterize the thermal properties of subwavelength objects. Moreover, these results clearly illustrated the limitations of Planck’s law when dealing with small objects. Actually, the quantitative explanation of the reported results is still open and it would be very interesting to see if fluctuational electrodynamics^14^, the modern theory that is supposed to describe all thermal radiation phenomena, can reproduce the experimental observations. Moreover, this technique could be used to study more complex systems, such as gratings or metamaterials, with the goal to make the thermal emission even more coherent.

## Super-Planckian far-field radiative heat transfer

A related breakthrough has been the recent discovery that the far-field radiative heat transfer (RHT) between two small objects can greatly overcome the black-body limit^6, 7^. While it is known that the thermal emission of a macroscopic object cannot overcome this limit^15^, no fundamental law prevents a subwavelength object from emitting more than a black body. In fact, the effective emissivity of a small object is given by its absorption efficiency (the absorption cross section divided by the geometrical one), and this quantity can be larger than one. However, to overcome the black-body limit requires a broadband resonant emission (with efficiencies larger than 1), something that is difficult to achieve in practice and, in fact, super-Planckian thermal emission is only expected in rather academic situations^12^. In the context of RHT between objects, the possibility of overcoming the Planckian limit in the far-field regime, i.e., when objects are separated by distances larger than *λ*_Th_, had until recently never been predicted or observed.

**Fig. 2:**
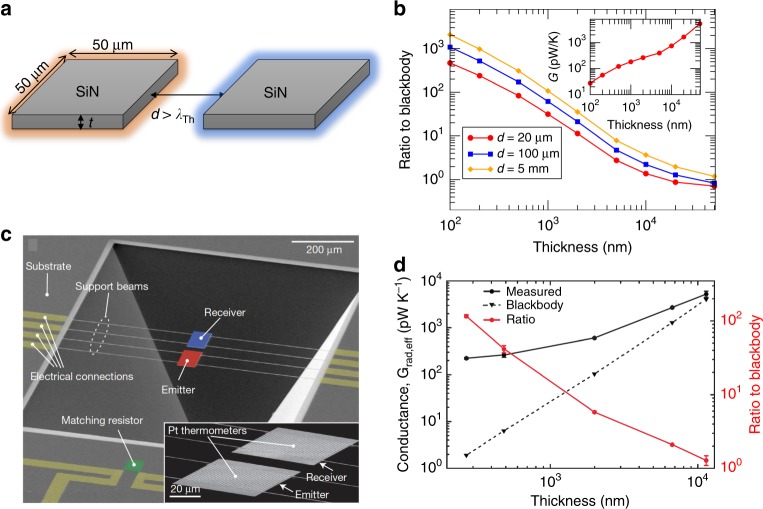
Super-Planckian far-field radiative heat transfer in SiN suspended pads. **a** SiN pads with lateral dimensions of 50 μm × 50 μm and thickness *t* separated by a gap *d* larger than the thermal wavelength *λ*_Th_. **b** Computed ratio between the exact radiative heat conductance and the blackbody result for the system in panel a as a function of the pad thickness and at room temperature^6^. The different curves corresponding to the different values of the gap in the far-field regime (see legend). The inset shows the absolute value of the thermal conductance for a 20 μm gap. Panel **a** and **b** were adapted with permission from ref. ^6^. Copyright (2018) by the American Physical Society. **c** Nanofabricated experimental platform used to probe radiative heat transfer^7^. The receiver and emitter devices are SiN pads suspended by support beams from a substrate and feature embedded Pt resistance thermometers-heaters. The pads have lateral dimensions of 60 μm × 80 μm and varying thickness. **d** Measured radiative conductance in the setup of panel **c** at room temperature (black circles; left axis) for a 20 μm gap as a function of the pad thickness from about 11 μm to 270 nm. The black triangles (left axis) correspond to the computed blackbody result and the red circles (right axis) are the ratio of the measured to the simulated radiative conductance. Notice that the back-body limit is overcome by more than two orders of magnitude for the thinnest devices. Panel **c** and **d** were reprinted from ref. ^7^ with permission. Copyright (2019) from Springer Nature

The situation changed when Fernández-Hurtado et al.^6^ predicted that the far-field RHT between objects with dimensions smaller than *λ*_Th_ can overcome the black-body limit by orders of magnitude. This conclusion was obtained within the framework of fluctuational electrodynamics^14^. Interestingly, it was found that the far-field RHT is determined by the directional absorption efficiencies of the individual objects. This relation suggests that the black-body limit can be overcome by using highly anisotropic systems with very directional thermal emission. In particular, Fernández-Hurtado et al.^6^ predicted that super-Planckian far-field RHT can take place between dielectric nanoribbons (made of SiO_2_ or SiN), similar to those studied by Chen and colleagues^8^, and in suspended-pad micro-devices also made of these polar dielectrics (Fig. 2a). The key is that when the thickness of these dielectric structures is reduced below *λ*_Th_, the absorption efficiencies become much larger than 1 over a broad range of frequencies simply by increasing the length of the structure. This is due to the fact that these structures behave as lossy dielectric waveguides that absorb the radiation very efficiently along their edges. In the case of suspended pads, this extraordinary absorption efficiency enables to overcome by black-body limit by several orders of magnitude when the device thickness is on the order of a few hundred nm, while the other two dimensions can be much larger than *λ*_Th_ (Fig. 2b).

Very recently, these ideas have been experimentally verified by Thompson et al.^7^ using SiN suspended-pad devices, which are normally used to measure the thermal transport through low-dimensional systems. In these devices, the SiN pads feature platinum resistors to control the temperature difference across the gap and to measure the heat transfer (Fig. 2c). These authors used SiN pads with lateral dimensions of 60 μm × 80 μm and a thickness ranging from 270 nm to 11.4 μm. The separation between the pads was equal or larger than 20 μm to be in the far-field regime. In agreement with the predictions^6^, they reported radiative heat conductance values up to two orders of magnitude larger than the black-body limit for the thinnest devices (Fig. 2d). They also showed that this super-Planckian RHT persists for a wide range of temperatures (100–300 K) and is still visible at macroscopic distances on the order of 1 mm. These observations further illustrated the limitations of Planck’s law to describe the thermal properties of micro- and nano-systems. They also showed that nanoscale systems may have extraordinary absorption properties that could be used for energy conversion applications. These results are also important for the study of the thermalisation of small objects^13^, which has implications, e.g., in cavity opto-mechanics^16^ or in the study of interstellar dust in astrophysics^17^.

## Outlook

We have just scratched the surface in understanding thermal radiation properties of subwavelength objects. For instance, the experimental techniques mentioned above are only able to study systems where some of the dimensions are still macroscopic. Thus, it would be highly desirable to develop techniques to probe, directly or indirectly, the radiative heat transfer or thermal emission of truly nanoscale objects. In fact, some progress has already been made in the context of levitated nanoparticles^16^. On the other hand, the theory has to keep up with the experimental advances and must be adapted to describe situations with complex temperature profiles and nontrivial thermalisation processes that take place in nanoscale objects^18^.
